# Wrist-Worn and Arm-Worn Wearables for Monitoring Heart Rate During Sedentary and Light-to-Vigorous Physical Activities: Device Validation Study

**DOI:** 10.2196/67110

**Published:** 2025-03-21

**Authors:** Theresa Schweizer, Rahel Gilgen-Ammann

**Affiliations:** 1 Department of Monitoring and Evaluation, Swiss Federal Institute of Sport Magglingen SFISM, Hauptstrasse 247, Magglingen, 2532, Switzerland

**Keywords:** validity, reliability, accuracy, wearable devices, wearing position, photoplethysmography, heart rate

## Abstract

**Background:**

Heart rate (HR) is a vital physiological parameter, serving as an indicator of homeostasis and a key metric for monitoring cardiovascular health and physiological responses. Wearable devices using photoplethysmography (PPG) technology provide noninvasive HR monitoring in real-life settings, but their performance may vary due to factors such as wearing position, blood flow, motion, and device updates. Therefore, ongoing validation of their accuracy and reliability across different activities is essential.

**Objectives:**

This study aimed to assess the accuracy and reliability of the HR measurement from the PPG-based Polar Verity Sense and the Polar Vantage V2 devices across a range of physical activities and intensities as well as wearing positions (ie, upper arm, forearm, and both wrists).

**Methods:**

Sixteen healthy participants were recruited to participate in this study protocol, which involved 9 activities of varying intensities, ranging from lying down to high-intensity interval training, each repeated twice. The HR measurements from the Verity Sense and Vantage V2 were compared with the criterion measure Polar H10 electrocardiogram (ECG) chest strap. The data were processed to eliminate artifacts and outliers. Accuracy and reliability were assessed using multiple statistical methods, including systematic bias (mean of differences), mean absolute error (MAE) and mean absolute percentage error (MAPE), Pearson product moment correlation coefficient (*r*), Lin concordance correlation coefficient (CCC), and within-subject coefficient of variation (WSCV).

**Results:**

All 16 participants (female=7; male=9; mean 27.4, SD 5.8 years) completed the study. The Verity Sense, worn on the upper arm, demonstrated excellent accuracy across most activities, with a systematic bias of −0.05 bpm, MAE of 1.43 bpm, MAPE of 1.35%, *r*=1.00, and CCC=1.00. It also demonstrated high reliability across all activities with a WSCV of 2.57% and no significant differences between the 2 sessions. The wrist-worn Vantage V2 demonstrated moderate accuracy with a slight overestimation compared with the ECG and considerable variation in accuracy depending on the activity. For the nondominant wrist, it demonstrated a systematic bias of 2.56 bpm, MAE of 6.41 bpm, MAPE 6.82%, *r*=0.93, and CCC=0.92. Reliability varied considerably, ranging from a WSCV of 3.64% during postexercise sitting to 23.03% during lying down.

**Conclusions:**

The Verity Sense was found to be highly accurate and reliable, outperforming many other wearable HR devices and establishing itself as a strong alternative to ECG-based chest straps, especially when worn on the upper arm. The Vantage V2 was found to have moderate accuracy, with performance highly dependent on activity type and intensity. While it exhibited greater variability and limitations at lower HR, it performed better at higher intensities and outperformed several wrist-worn devices from previous research, particularly during vigorous activities. These findings highlight the importance of device selection and wearing position to ensure the highest possible accuracy in the intended context.

## Introduction

Heart rate (HR) is one of the most commonly measured physiological parameters in wearables, valued for its ease of measurement and its role as a key marker of homeostasis, cardiovascular health, and physiological responses. HR can provide early warnings for certain pathological conditions; for example, resting HR is an independent predictor of cardiovascular disease, stroke, and sudden death [1,2]. In addition, HR is frequently used for assessing physical effort, workload intensity, and supporting performance monitoring. It is also often integrated into algorithms to estimate other physiological metrics, such as core body temperature and energy expenditure [3-5]. HR is therefore a valuable and valid parameter when aiming for health monitoring and workload management.

The current criterion measure for assessing HR outside the laboratory is the chest strap, which uses electrocardiogram (ECG) technology, due to its strong agreement and minimal bias when compared with the ECG-Holter device in healthy adults and patients [6-10]. A prior validation study demonstrated that the Polar H10 (H10; Polar Electro Oy) exhibited even higher accuracy during higher-intensity activities with increased motion than the ECG-Holter [11]. However, the continuous use of chest straps every day in the field can lead to discomfort, incompatibility with equipment, or displacement issues [12]. Consequently, there is growing interest in wrist-, upper arm-, or forearm-wearable devices, which use photoplethysmography (PPG) [13]. PPG is a noninvasive measurement technique that detects blood volume changes in the microvascular bed of tissue by illuminating the skin and measuring the reflected light [14].

The affordability and capability of these wearable devices to continuously monitor physiological parameters over extended periods, combined with rapid advancements in multimodal sensing technologies and extensive marketing by manufacturers, have led to their widespread use. However, the quality of the data is crucial when monitoring health parameters in real life. Many users—and even scientists—may rely on these devices to measure outcomes such as resting HR, training zones, fatigue, or health issues without verifying the accuracy and reliability of the measured physiological parameters. Notably, one critical review showed that more than half of the technologies reviewed had not been validated through independent research, with only 5% having been formally validated [13]. As wearable technologies continue to evolve with each update or new version including new sensor modalities, it is important to conduct ongoing assessements of their accuracy and reliability, as these factors can impact measurement performance [1,15-18].

Furthermore, validation studies often focus on only 1 or a few standardized exercises (eg, resting, cycling, or treadmill running) that involve minimal movement artifacts in the arms or wrists and are conducted in controlled laboratory settings [19-21]. In fact, HR measurement accuracy has shown to be influenced by differences in blood flow, motion artifacts, and the interaction between the sensor and skin on the different wearing position [22-25]. For example, proximal wearing position such as the upper arm may provide more stable readings during high-motion activities than distal placements such as the forearm or the wrist, where movement artifacts are more pronounced and blood flow is lower. For HR monitoring to be applicable to general activity tracking, data should be validated across a variety of exercise modalities at different intensities (resting, submaximal, and high) and body positions (lying, sitting, and standing), as well as during free movement [15].

Although the H10 is recognized as a criterion measure based on the INTERLIVE Network’s expert statement [26], the Polar Verity Sense (Polar Electro Oy) offers a possible alternative. When worn on the upper arm, the Verity Sense sits well on the skin, may be less intrusive than a chest strap, and provides advantages over a wrist-worn device due to its proximal wearing position (eg, increased blood flow). The Verity Sense has been evaluated in prior studies, though the activities were in some of the studies very short, laboratory-based, in paced conditions, or very specific (eg, walking, jogging, swimming, Pickleball Game Play, or biking) [27-31]. Similarly, the Vantage V2 has been validated in prior studies, but the studies had either an older criterion measure or was validated in specific activities in laboratory conditions (eg, paced running and swimming) [31-33]. To the authors’ knowledge, no study has evaluated the different wearing locations and tested it in various types of exercises and intensities in a more naturalistic environment.

Therefore, this study aims to validate the Polar Verity Sense and Vantage V2 in terms of HR across diverse activities, intensities, and wearing positions in conditions that closely resemble free-living environments over a sufficient amount of time to get robust results. The study incorporates a variety of activities, including different resting (eg, lying and sitting), common exercises (eg, running and cycling), body weight exercises, and dynamic movements such as parkour, which introduce significant challenges such as variations in blood flow and involve high levels of motion. To ensure robust findings, the protocol will be repeated twice to assess the reproducibility of HR measurements.

## Methods

### Participants

Sixteen healthy participants were recruited for this study. Recruitment was conducted via email announcements and in-person assessments of students and staff at the Swiss Federal Institute of Sport Magglingen. The study aimed to include individuals with diverse fitness levels and training habits, ensuring representation of both those who met and those who did not meet the World Health Organization’s recommendation of 150‐300 minutes of moderate-intensity aerobic physical activity per week [34]. Participants had to be between 18 and 40 years of age with a BMI between 18.5 and 30 kg/m². Interested participants received detailed study information and provided written informed consent before participation. Prior to inclusion, they were screened using the Physical Activity Readiness Questionnaire to ensure that they met the eligibility criteria. Only those who answered “no” to all Physical Activity Readiness Questionnaire questions, did not take any medication affecting HR, had no known ECG abnormalities, and had no tattoos on the sensor placement areas (upper arms, forearms, and wrists) were included in the study. In addition, skin type was assessed using the Fitzpatrick Scale [35], and the amount of body hair on the wrists and arms was recorded.

### Experimental Procedure

The participants were tested individually on different days and at different times of the day. The measurements were conducted in a gymnasium with prepared areas to perform the different activities and with consistent environmental conditions, with a mean (SD) ambient temperature of 19.5 °C (SD 0.9 °C) and humidity of 49.8% (SD 3.9%). After recording each participant’s weight, height, skin color, and body hair (while they were dressed in underwear), all devices were placed in the specific wearing positions on the body as recommended by the manufacturers. The H10 chest strap was moistened prior to use. All devices were activated at least 5 minutes before the protocol began to allow the sensors to calibrate to the HR.

**Figure 1.**
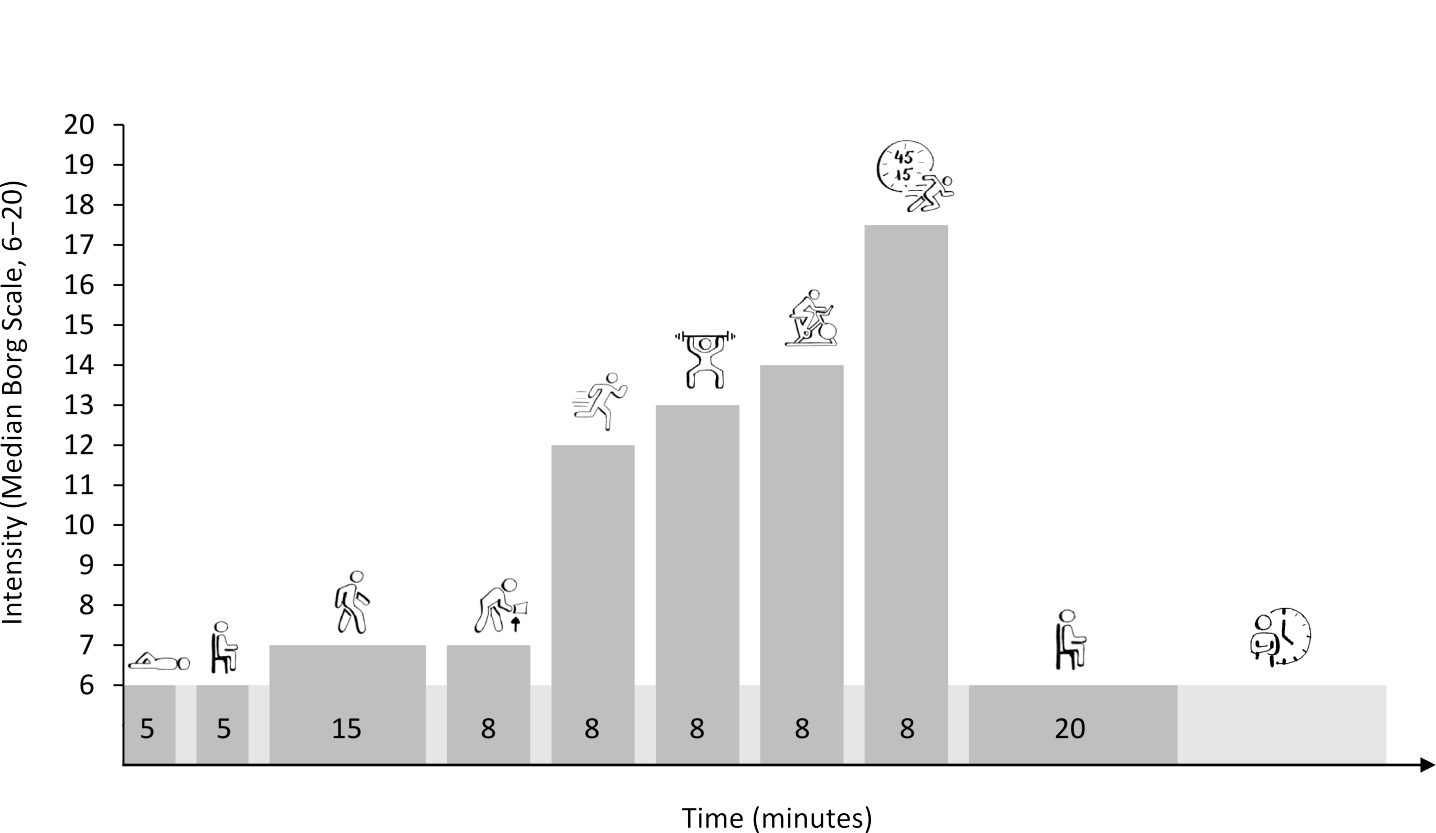
Study protocol with 9 activities with 2-minute breaks in between. This protocol was repeated twice with a 20-minute break between sessions. Lower-intensity activities, such as lying down, sitting, and postexercise sitting, showed a median (IQR) rating of perceived exertion (RPE) of 6.0 (1.0), indicating minimal exertion. Low-intensity activities, including walking and picking up objects, had RPE values of 7.0 (1.25) and 7.0 (2.0), respectively, while jogging and weight training had RPE values of 12.0 (2.25) and 13.0 (2.0). Higher-intensity activities, such as cycling and high-intensity interval training, had median RPEs of 14.0 (2.25) and 17.5 (2.0), respectively, the latter reflecting near-maximum exertion. Across all activities, the median RPE was 10.0 (7.0).

The study protocol consisted of 9 different activities in order of increasing intensity (Figure 1): lying down (5 minutes), sitting (5 minutes), walking (15 minutes), picking up objects (8 minutes), jogging (8 minutes), weight training (8 minutes consisting of squats, biceps curls, lunges, and abdominal crunches), cycling on an ergometer (8 minutes), high-intensity interval training (HIIT; 8 minutes of a continuous parkour containing sprinting, dragging, carrying, lifting, and hammering, with 45 seconds of effort and 15 seconds of rest), and postexercise sitting (20 minutes). A 2-minute rest was taken between activities, and the entire protocol was repeated twice, with a 20-minute break between sessions in which the participants sat down, rested, and could drink or eat something, if needed. The procedures and instructions were standardized and identical for all participants, but they were kept very short to enhance the naturalistic study design. The participants rated their exertion using the Borg Rating of Perceived Exertion scale (6‐20) after each activity to quantify intensity levels, ranging from minimal to near-maximal exertion [36,37].

### Devices and Instruments

#### Wearable Devices

The Polar H10 (H10) measures HR using 1-lead ECG technology with a sampling frequency of 1000 Hz. According to the INTERLIVE Network’s expert statement, ECG chest straps that have been independently validated and demonstrate excellent agreement with respect to beats per minute (ie, >95%) are considered appropriate criterion measures for evaluating wearable technologies measuring HR [26]. The H10 is included in their list of validated devices, with a prior study showing an excellent agreement (*r*=0.997) and 97.1% of the measured RR intervals (ie, time between successive R-wave peaks in the QRS complex—a waveform in an ECG representing ventricular depolarization and contraction, which corresponds to one full cardiac cycle) differing by less than 2% during various activities and intensities [11].

**Figure 2.**
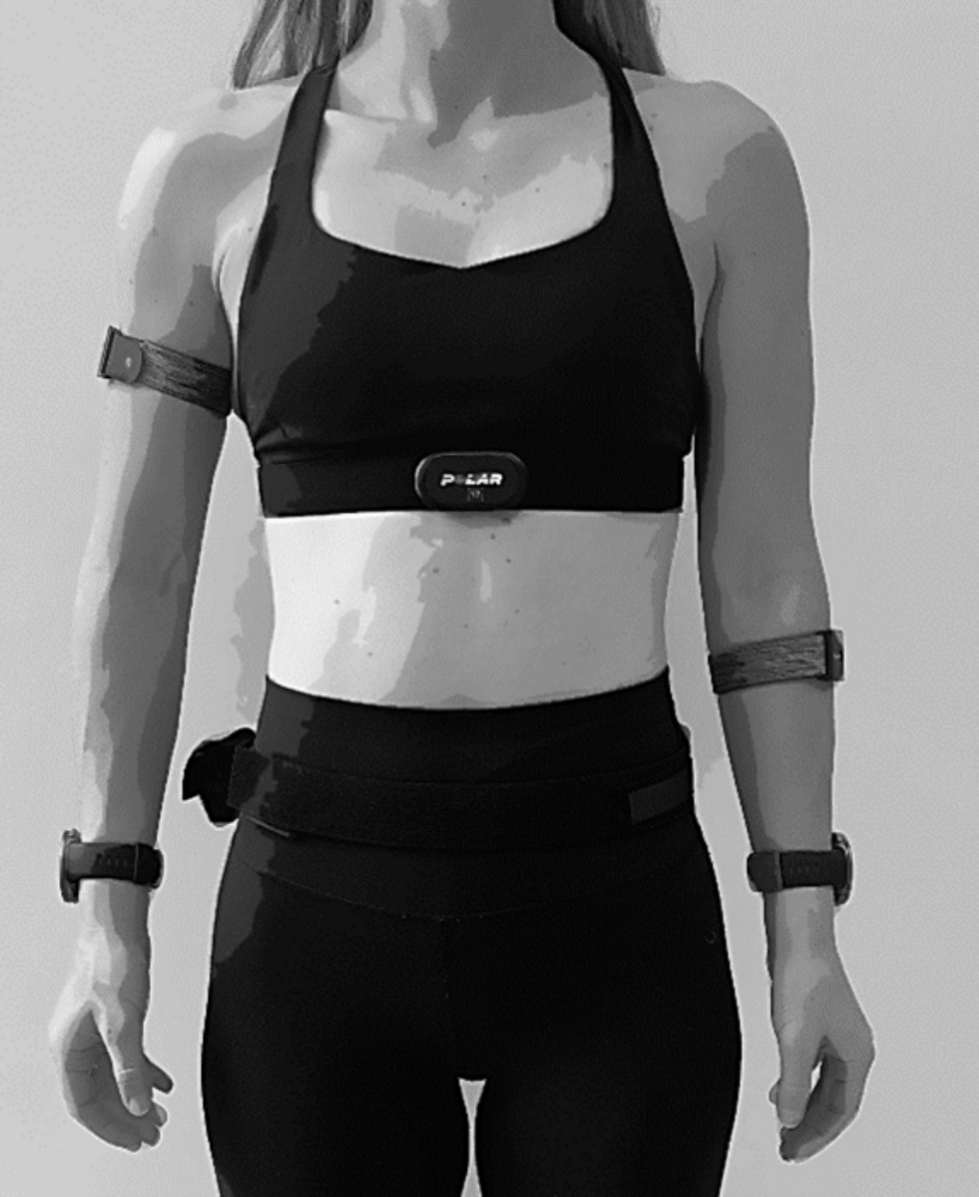
Placement of the different wearable devices. The H10 chest belt was placed on the chest with a Vantage V2 as logger on the waist. A Vantage V2 was placed on each wrist. A Verity Sense was placed on the upper arm and forearm.

In this study, 2 wearable devices were evaluated. Both were placed on different wearing positions. The Verity Sense (Polar Electro Oy) measures HR on the upper arm and forearm using optical PPG technology with a sampling frequency of 1 Hz (firmware version: 2.0.3). The Vantage V2 (Polar Electro Oy) measures HR on the wrist using optical PPG technology with a sampling frequency of 1 Hz (firmware version: 4.1.0). Figure 2 shows the devices included in the study as well as their positions on the body. The Verity Sense devices were placed on the forearm and upper arm of opposite sides, with the specific side (left or right) randomly assigned across participants. Two Vantage V2 watches were placed on the wrists of each participant to capture readings from both the dominant and nondominant sides. One more Vantage V2 was used as a data logger for the H10 and placed in a small pocket on an elastic belt around the waist. The Vantage V2 were started in the activity mode “other indoor” as no Global Positioning System was needed and different activities were performed. The Verity Sense were started in “recording mode”. All data were downloaded from the web-based Polar Flow application (Polar Electro Oy).

#### Other Instruments

The body heights of the participants were measured using a stadiometer (model 214; Seca GmbH), and body weight was measured on a calibrated digital balance scale (model 877; Seca GmbH). The cycling ergometer Ergoselect 200 (Ergoline GmbH) was used for the cycling activity, and dumbbells weighing from 2.5 to 10 kg were used for the weight training. A weather station was used to measure ambient temperature and humidity.

### Data Processing and Cleaning

First, all rest periods between activities were removed from the data. Second, the HR data derived from the PPGs (Verity Sense and Vantage V2) were synchronized with the reference using time stamps from the exported file and cross-correlated to fix the inconsistent lags between the ECG- and PPG-derived HR signals [38,39]. Third, missing values (ie, blanks or zeros) and artifacts were quantified. Data were considered artifacts if they fell below 30 bpm (type I), if they exceeded 230 bpm (type II), or if consecutive values differed by 15 bpm (type III) [40,41]. All artifacts were then removed from the dataset. Fourth, all reference data from the H10 device were statistically and visually inspected for potential outliers or irregularities to prevent errors from being mistakenly attributed to the Verity Sense and Vantage V2 devices. For each participant, the activities were flagged if they contained more than 10 missing data points, more than 10 artifacts, or a Pearson correlation below 0.9 compared with the Verity Sense or Vantage V2. The flagged activities underwent further visual screening to identify whether the error originated from the H10. If the H10 data contained a substantial number of outliers or were considered irregular, the entire activity was excluded from the analysis. Finally, HR data were averaged in 10-second intervals for each activity.

### Statistical Analysis

Statistical analysis was performed in accordance with previous recommendations [15]. The data from the tested devices and the criterion measure were assessed for normality, and all data were found to be normally distributed.

Accuracy was assessed for overall data and for each activity using systematic bias (mean of differences) with 95% limits of agreement (LoA), accompanied by the results of a 2-tailed 1-sample *t* test performed on the differences between the 2 measurements (ie, difference from zero). Moreover, mean absolute error, mean absolute percentage error (MAPE), 5% accuracy (percentage of MAPE within a 5% range of the reference value), root-mean-squared error (RMSE), and ordinary least squares linear regression were used to evaluate accuracy. Although previous validation studies lack consensus and have defined varying accuracy thresholds, this study classified a device as having very high accuracy if MAPE was <3%, high accuracy if MAPE was <5%, and moderate accuracy if MAPE was <10%, based on criteria used in some validation studies [21,28,31,42,43]. Pearson product moment correlation coefficient (*r*) and Lin concordance correlation coefficient (CCC) were used to evaluate the agreement between the criterion measure and the wearable device [44-46]. The Pearson correlation coefficient was interpreted as follows: 0.45‐0.69 (very poor), 0.70‐0.84 (poor), 0.85‐0.94 (good), 0.95‐0.994 (very good), and >0.995 (excellent) [47]. The strength-of-agreement criteria for the CCC were interpreted using McBride’s (2005) criteria: <0.90 (poor agreement), 0.90‐0.95 (moderate agreement), 0.95‐0.99 (substantial agreement), and >0.99 (almost perfect agreement) [44].

Reliability was assessed using the within-subject coefficient of variation (WSCV), calculated based on the differences between the tested devices and the reference data, where lower values indicate greater consistency. Based on a prior study, the threshold of <5% was used to indicate high reliability, while <10% was considered acceptable reliability [21]. In addition, reproducibility was assessed using the Wilcoxon signed rank test to compare the differences between the device and reference measurements between session 1 and session 2. All data processing, cleaning, and analysis was done with Python (version 3.12; Python Software Foundation).

### Ethical Considerations

This study involving human participants was reviewed and approved by the Swiss ethics committee (project ID: 2022‐01456). The research design adhered to the ethical standards outlined in the Declaration of Helsinki. All data collected were deidentified to ensure participant confidentiality. No personal identifiers were included in the dataset, and access to raw data was restricted to authorized researchers only. Participants provided written informed consent, which included permission for their anonymized data to be used in publications and shared with other researchers for further research purposes, in strict adherence to data protection regulations. Participants received a gift card valued at 30 Swiss Francs (CHF), approximately US $29 based on the exchange rate at the time of the study, as compensation for their time and participation. No identifiable images of participants are included in the manuscript or supplementary materials.

## Results

### Participants

Sixteen healthy participants (female=7; male=9; dominant right-handed=13) volunteered for this study. Their demographic characteristics reported as mean (SD) were age: 27.4 (5.8) years, height: 173.5 (9.2) cm, weight: 69.9 (9.4) kg, and BMI: 23.1 (2.0) kg/m². Ten participants met the recommendations of the World Health Organization of 150‐300 minutes of moderate-intensity aerobic physical activity per week and 6 were below that threshold. Six participants were classified as type I, and 10 participants were classified as type II according to the Fitzpatrick Scale. In addition, none of the participants had exceptionally hairy skin at any of the device-wearing positions.

### Missing Values, Artifacts, and Outliers

No devices had missing values; however, artifacts and outliers were identified in the H10 and Verity Sense data. For the H10, 9 randomly occurring type III artifacts were found. In addition, visual screening led to the overall removal of 16,462 seconds (10%) of the raw data from 3 participants, including the entire protocol’s first session of 1 participant and the second session of 2 participants. These outliers were potentially due to suboptimal positioning or displacement of the H10 in these 3 participants. In the Verity Sense data, 85 seconds (0.06%) were classified as type I artifacts (upper arm: 36; forearm: 49) and 32 seconds (0.02%) as type III artifacts (upper arm: 3; forearm: 29). No specific activity, participant, or gender could be identified as having more artifacts than the others.

After averaging the cleaned data into 10-second intervals, the data from the 16 participants totaled 40.7 hours (mean 4.5, SD 2.1 hours per participant), resulting in 14,653 10-second data points analyzed across all activities. The sedentary or resting activities, including lying down, sitting, and postexercise sitting, contributed 867, 870, and 3346 data points, respectively, totaling 5083 (34.7%) data points. Low- to moderate-intensity activities, such as walking and picking up objects, provided 2610 and 1392 data points, respectively, amounting to 4002 (27.3%) data points. Higher-intensity activities, including jogging, weight training, cycling, and HIIT, each contributed 1392 data points, for a total of 5568 (38.0%) data points. This distribution ensured comprehensive coverage across all activity types and intensities.

### Accuracy and Reliability

#### Arm-Worn Verity Sense

The overall mean bias was −0.05 bpm (LoA –5.84 to 5.74 bpm) on the upper arm and −0.91 bpm (LoA –14.64 to 12.83) on the forearm, indicating only minimal underestimation of the HR measurements. The 2-tailed 1-sample *t* test was conducted to determine whether the differences between the Verity Sense and the reference measurement significantly deviated from zero. The results indicated no significant difference on the upper arm for lying (*P*=.845), sitting (*P*=.093), jogging (*P*=.159), and postexercise sitting (*P*=.911). Likewise, on the forearm, no significant differences were found for lying (*P*=.981), walking (*P*=.227), and jogging (*P*=.306). No significant differences were found overall and for all other activities (*P*<.05). For the upper arm placement, MAPE remained low across all activities, with the lowest values observed during jogging (0.69%) and cycling (0.53%) and the highest during sitting (2.48%) and picking up objects (2.34%). On the forearm, MAPE was slightly higher overall, with the lowest values recorded during jogging (0.92%) and cycling (0.60%). The overall 5% accuracy was 95% for the upper arm and 89% for the forearm. The RMSE for the upper arm was generally low across activities, with an overall value of 2.95 bpm, except for weight training, which showed an RMSE of 6.49 bpm. RMSE values for the forearm were higher, with an overall mean of 7.07 bpm. Pearson correlation coefficients demonstrated very good to excellent positive linear correlations between the Verity Sense and the ECG criterion across all activities for the upper arm (*r*>0.94). For the forearm, the correlations similarly ranged from very good to excellent for all activities (*r*>0.95), except weight training (*r*>0.88), HIIT (*r*>0.85), and postexercise sitting (*r*>0.79). Regression analyses supported these findings, with strong correlations (*r*²=0.99 for the upper arm and *r*²=0.96 for the forearm) and regression slopes near 1.00, especially during lower-intensity activities, except for weight training. The CCC showed consistently almost perfect agreement, with an overall CCC of 1.00 (95% CI 0.99-1.00) for the upper arm, although lower values were observed during weight training. For the forearm, the CCC showed substantial agreement with an overall value of 0.98 (95% CI 0.97-0.98), with decreased agreement during HIIT and postexercise sitting.

The Verity Sense demonstrated high reliability across most activities, regardless of arm placement. The Wilcoxon signed rank test showed no significant differences between the device and reference measurements across sessions for the upper arm (*W*=2994.0, *P*=.213; session 1: mean_diff_ –0.14 bpm, SD_diff_ 0.87 bpm; session 2: mean_diff_ –0.07 bpm, SD_diff_ 1.70 bpm) and forearm (*W*=3081.0, *P*=.314; session 1: mean_diff_ –0.61 bpm, SD_diff_ 2.63 bpm; session 2: mean_diff_ –1.06 bpm, SD_diff_ 5.74 bpm) placements. In addition, the WSCV was consistently low, particularly for the upper arm (ranging from 0.98% for cycling to 4.98% for weight training), while the forearm exhibited slightly higher variability (1.14% for cycling to 9.80% for postexercise sitting).

Table S1 in Multimedia Appendix 1 shows the detailed accuracy and reliability results for the Verity Sense compared with the reference for each activity and for each wearing position.

#### Wrist-Worn Vantage V2

The overall mean bias was 2.93 bpm (LoA –20.46 to 26.31) and 2.56 bpm (LoA –21.88 to 26.99) for the dominant and nondominant wrists, respectively, indicating a slight overestimation of HR with large LoAs. For the 2-tailed 1-sample *t* test, for both the dominant and nondominant wrists, no significant difference was found for sitting (*P*=.271; *P*=.818), whereas all other activities showed significant differences (*P*<.001).

For both wearing positions (dominant and nondominant), MAPE was lowest during jogging (3.84% and 3.55%), cycling (1.17% and 2.06%), and postexercise sitting (2.15% and 2.07%). However, MAPE exceeded 10% during activities characterized by lower HR, such as lying down, walking, and picking up objects. The 5% accuracy showed varying levels of agreement across all activities, with an overall result of 73.56% for the dominant wrist and 71.83% for the nondominant wrist. For both the dominant and nondominant wrists, RMSE was generally high, with overall values of 12.29 bpm and 12.73 bpm, respectively. However, accuracy improved during postexercise sitting, where RMSE was lower at 3.60 bpm and 3.78 bpm. Pearson correlation and regression analyses further highlighted these discrepancies. For both the dominant and nondominant wrists, correlation was good to very good during jogging (*r*=0.89 and *r*=0.91), weight training (*r*=0.90 and *r*=0.91), cycling on an ergometer (*r*=0.98 and *r*=0.94), and postexercise sitting (*r*=0.97 and *r*=0.97). However, accuracy was very poor to poor for all other tasks. A slight difference between wearing positions was observed during HIIT, where the dominant wrist showed poor correlation (*r*=0.81), while the nondominant wrist showed good correlation (*r*=0.85). In addition, linear regression slopes indicated overall low agreement, with values of 0.87 and 0.85 for the dominant and nondominant wrists, respectively. On the dominant wrist, CCC ranged from poor agreement (0.25 during picking up objects) to substantial agreement (0.97 during cycling). On the nondominant wrist, CCC values ranged from poor agreement (0.24 during picking up objects) to substantial agreement (0.97 during postexercise sitting).

The Vantage V2 demonstrated moderate reliability across most activities for both wrist placements. The Wilcoxon signed rank test showed no significant differences between the device and reference measurements across sessions for the dominant wrist (*W*=3379.0, *P*=.844; session 1: mean_diff_ 3.72 bpm, SD_diff_ 10.96 bpm; session 2: mean_diff_ 3.63 bpm, SD_diff_ 10.32 bpm) and the nondominant wrist (*W*=2852.5, *P*=.103; session 1: mean_diff_ 3.51 bpm, SD_diff_ 12.37 bpm; session 2: mean_diff_ 2.41 bpm, SD_diff_ 8.73 bpm). Although no significant differences were found between sessions, the WSCV varied across activities. Lower variability was observed for postexercise sitting (3.49% on the dominant wrist; 3.64% on the nondominant wrist), while very high variability was found during lying down (26.44% on the dominant wrist; 23.04% on the nondominant wrist). Overall, variability remained high, with overall WSCV values of 10.41% for the dominant wrist and 10.87% for the nondominant wrist.

Table S1 in Multimedia Appendix 2 shows the detailed accuracy and reliability results for the Vantage V2, compared with the reference for each activity and for each wrist placement.

## Discussion

### Principal Findings and Comparison With Prior Work

#### Arm-Worn Polar Verity Sense

This study evaluated the accuracy and reliability of the arm-worn Verity Sense across various activities and both placements, the forearm and the upper arm. The device had no missing values and only a trivial number of artifacts (0.08%). Overall, and especially on the upper arm, the Verity Sense demonstrated minimal bias (−0.05 bpm), very high accuracy (MAPE 1.35%), and very good to excellent agreement with ECG (*r*=1.00, CCC 1.00). Reliability was also high, with no significant differences between sessions and consistently low variability in comparison with the criterion measure (WSCV 2.57%).

The overall trend suggested the highest accuracy and reliability during activities with elevated mean HR and less arm movements, while slightly lower accuracy was noted during low-intensity tasks such as weight training and object picking. As PPG-based HR measurements are influenced by differences in blood flow and motion artifacts, these findings underline the possible loss of accuracy with increased motion as well as reduced lower blood flow (eg, lower HR, cold extremities, and blood flow restriction due to clothes or other devices) [22-25]. These results align with previous studies that reported reduced accuracy in similar low-intensity, high-motion activities [16,28,31]. Notably, even during these challenging tasks, the upper arm placement continued to deliver strong results.

To the authors’ knowledge, regardless of the wearing position on the upper arm or the forearm, the excellent accuracy demonstrated by the Verity Sense in this study outperformed all of the following wearable devices tested in different activities and settings in previous studies: multiple Garmin wrist-worn devices (eg, Instinct, Venu, and Fenix 5‐6) [20,27,28,32,33,48,49], various Polar wrist-worn devices and the OH1 (ie, the prior version of the Verity Sense) [21,27,28,30,32,48], the Apple Watch [20,49], the Motiv Ring, the arm-worn Scosche Rythm+, the Jabra Elite Sport and the Suunto Spartan Sport [20], FitBit Charge 2 and 4 [19,43,50], and the Samsung Galaxy Watch Active2 [43].

In addition, in this study, the Verity Sense outperformed its own previous results from studies conducted between 2022 and 2024, demonstrating better MAPE values while maintaining similar regression analysis and CCCs [27-31,48]. These results suggest that the Verity Sense is a highly accurate and reliable alternative to the ECG-based chest strap such as the Polar H10. Notably, given the number of missing values and artifacts observed in the H10 in this study, the Verity Sense may offer greater robustness across the investigated activities. However, this study does not provide conclusive evidence of interchangeability between these devices.

#### Wrist-Worn Polar Vantage V2

This study evaluated the accuracy and reliability of the wrist-worn Vantage V2 across various activities and both wrist placements (dominant and nondominant). The device had no missing values or artifacts, suggesting a robust filtering method, as wrist-worn devices typically experience significant motion artifacts and low blood flow [22-25]. The Vantage V2 performed similarly on both wrists, showing a slight HR overestimation with large LoAs and overall moderate accuracy. However, accuracy varied considerably depending on the activity. High accuracy (MAPE<5%) was observed in all moderate- to vigorous-intensity activities (ie, jogging, weight training, cycling, and HIIT) as well as postexercise sitting, whereas activities with lower HR and increased motion artifacts exhibited poorer accuracy. Overall, although CCC demonstrated moderate agreement, Pearson correlation indicated good agreement and reached very good agreement during cycling on an ergometer and postexercise sitting, the 2 activities with low arm and wrist movement as well as increased blood flow. However, it is important to note that high correlations do not guarantee the absence of bias or error, nor do they confirm perfect validity [51]. Although no significant differences between sessions were found, overall reliability was below the acceptable threshold, with WSCVs exceeding 10%. Variability was particularly high during low-intensity activities (eg, lying down and picking up objects). In contrast, high to very high reliability was observed again during cycling on an ergometer and postexercise sitting. This again highlights the influence of motion artifacts combined with lower HR (ie, blood flow) on signal quality at the wrist position.

In previous studies, wrist-worn devices showed similar results: the bias tends to increase with the intensity of activity on a treadmill, while using a cycle ergometer, and during resistance training tasks [19,42,48,49,52,53]. Similarly, one study found that the magnitude of the errors depended on the activity type and that it can result in an absolute error that is 30% higher than at rest [38]. Wrist-worn devices are more susceptible to noise and distortion due to thinner skin, underlying bones and tendons, and reduced blood perfusion, all of which increase the likelihood of motion artifacts in wrist-worn devices compared with arm-worn devices [24]. Moreover, arm and wrist movements cause displacement of the PPG sensor over the skin, alter skin deformation, and affect blood flow dynamics, generating motion artifacts that are difficult to mitigate through filtering or algorithms when occurring frequently and result in false calculations [22,25]. Although the Vantage V2 also uses PPG technology, like the Verity Sense, the difference in wearing position has a great impact on the HR signal quality, requiring distinct filtering methods and algorithms. Similarly, since wrist-worn devices measure at a more distal position, blood flow may be further reduced in cold environments due to vasoconstriction, which has a greater impact on smaller capillaries in the extremities than in the upper arm. Moreover, a good fit on the wrist plays a crucial role in minimizing device movement on the skin, which in turn reduces skin deformation.

In this study, the Vantage V2 performed best during cycling on an ergometer, contrary to the expectation that wrist posture during cycling might negatively impact accuracy [19]. This improved performance could be attributed to ensuring a proper fit of the watch, with the device positioned correctly above the wrist and snugly fitted, which might mitigate issues caused by wrist bending.

Notably, the Vantage V2 showed similar results to, or even outperformed, other wrist-worn devices evaluated in previous studies, particularly during higher-intensity activities. When compared with similar current devices, such as the Garmin Forerunner 945 and Polar Ignite, the Vantage V2 demonstrated slightly higher or similar mean absolute error and MAPE values but exhibited comparable LoAs and slightly stronger positive correlations [54]. In low-intensity activities such as walking, the Vantage V2 showed lower accuracy (ie, higher MAPEs) than the Polar Vantage M and the Garmin Instinct. However, during higher-intensity activities such as jogging and skipping (comparable with HIIT), the Vantage V2 outperformed both devices [28]. During lying, sitting, walking, and squat training (which can be compared with weight training in this study), the Vantage V2 exhibited higher MAPEs in lying and walking but lower MAPEs in sitting and weight training compared with the Fitbit Charge 4 and Samsung Galaxy Watch Active2 [43]. Similarly, in terms of agreement (Pearson correlation), the Vantage V2 exhibited lower agreement in low-intensity activities but outperformed the Apple Watch Series 4, the Polar Vantage V, the Garmin Fenix 5, and the Fitbit Versa at higher HRs [33]. A comparable trend was observed when comparing the Vantage V2 with the Garmin Fenix 6 and the Polar Grit X across various moderate to vigorous activities (eg, walking, incremental maximal treadmill walking, and cycling) [48]. Furthermore, during cycling and resistance training, the Vantage V2 outperformed both the Apple Watch Series 2 and the Bose SoundSport Pulse [42]. The Vantage V2 also showed similar results to those of another study that tested this device in swimming [32].

These findings suggest that the Vantage V2 performs slightly better than its competitors at higher intensities and elevated mean HR, potentially indicating that the device incorporates a robust motion artifact filtering algorithm. However, it remains susceptible to lower blood flow. In summary, while the Vantage V2 still exhibits the typical limitations of wrist-worn sensors, its accuracy is comparable with—or even exceeds—that of some other wrist-worn devices.

### Strengths, Limitations, and Recommendations

This study has several strengths but also faces certain limitations that warrant consideration. First, while the sample size was relatively small and homogeneous in terms of health, age (mean 27.4, SD 5.8 years), and BMI (18.5‐30 kg/m²), the study benefited from a large dataset (14,653 data points; mean 4.5, SD 2.1 hours per participant). This extensive data volume strengthens the reliability of the analysis and allows for robust analysis. Future research should complement this approach by including a more diverse population to assess broader applicability. Second, the study protocol included a wide range of activities, from sedentary to vigorous intensity, conducted in seminaturalistic conditions in a gymnasium. However, the indoor environment may not fully replicate real-world conditions, and activities outside this range, such as extreme sports or water-based activities, were not evaluated. Third, while the Polar H10 ECG chest strap is a proven criterion measure for HR measurement during various activities and intensities, especially in free-living conditions, the H10 nevertheless exhibited missing data and artifacts in this study, potentially due to suboptimal sensor-wearing position or fitting, or motion-induced signal interference. To mitigate this, rigorous data cleaning and artifact detection procedures were used, including visual screening and the exclusion of outlier activities from the analysis. However, some artifacts may still have introduced variability into the reference data, potentially influencing the comparison with the tested wearable devices. Future studies should be aware of this limitation and carefully review the reference data as well, as errors or artifacts in the reference measurements could lead to misleading comparisons and affect the validity of the findings. Fourth, while the wearing position and fitting of the devices were standardized to ensure consistency, it might not reflect real-world usage where users may wear devices loosely or incorrectly. Including scenarios with varied placement conditions in future studies could better simulate real-world use. Furthermore, device placement on different limbs or at varying positions on the same limb may introduce variability due to differences in blood flow, which was not addressed in this study. Future research should explore whether placing an additional sensor on the same limb influences blood flow and, consequently, HR measurements. Finally, as wearable technologies continue to evolve, continuous validation across various activities, contexts, and populations will be crucial to ensuring that these devices provide accurate and actionable data for health monitoring and the development of physiological metrics (eg, estimation of core body temperature or energy expenditure).

### Conclusions

This study evaluated the accuracy and reliability of 2 currently available wearable devices across a wide range of activities and different wearing positions. The Polar Verity Sense demonstrated excellent accuracy and reliability across a broad range of physical activities and intensities, particularly when worn on the upper arm. The Polar Vantage V2, worn on the wrist, showed overall moderate accuracy and increased variability. It also demonstrated the typical limitations of wrist-worn devices, including reduced accuracy at lower HRs in combination with arm and wrist movements. However, it demonstrated improved performance at higher intensities and remains a competitive option within its category. These findings highlight the challenges associated with wrist-worn HR devices and the importance of device-wearing position to ensure accurate HR measurements.

In summary, for users seeking valid and reliable HR monitoring across various activities, the Verity Sense presents a strong alternative to ECG-based chest straps. For practical implementation, device selection should be guided by the intended use case, required accuracy, and user needs. Optimizing the chosen device and wearing position is essential to ensuring the highest possible accuracy within its specific context.

## Supplementary material

10.2196/67110 Multimedia Appendix 1 Accuracy and reliability results of the arm-worn Verity Sense (upper arm and forearm).

10.2196/67110 Multimedia Appendix 2 Accuracy and reliability results of the wrist-worn Polar Vantage V2 (dominant and nondominant wrists).

## Abbreviations

CCC: concordance correlation coefficient
ECG: electrocardiogram
HIIT: high-intensity interval training
HR: heart rate
LoA: limits of agreement
MAE: mean absolute error
MAPE: mean absolute percentage error
PPG: photoplethysmography
RMSE: root-mean-square error
WSCV: within-subject coefficient of variation
