# Supplementary material for: Wrist-Worn and Arm-Worn Wearables for Monitoring Heart Rate During Sedentary and Light-to-Vigorous Physical Activities: Device Validation Study
Source: JMIR Cardio. 2025 Mar 21;9:e67110. doi: 10.2196/67110 (PMC11951816; doi:10.2196/67110)
Supplement: Multimedia Appendix 1 [file cardio-v9-e67110-s001.docx]

**Table S1.** Accuracy and reliability results of the arm-worn Verity Sense (upper arm and forearm).

| **Verity Sense wearing position** | **Activity** | **HR**  **H10 (reference)  mean (SD) [bpm]** | **HR**  **Verity Sense mean (SD) [bpm]** | **Systematic bias  (LoA lower; LoA upper) [bpm]** | **MAE (MAPE) [bpm]** | **5% accuracy [%]** | **RMSE**  **[bpm]** | **Pearson’s correlation coefficient or OLS  *r*-value (*r*^2^)** | **OLS**  **slope**  **(intercept [bpm])** | **CCC (95% CI)** | **WSCV (95% CI)**  **[%]** |
| --- | --- | --- | --- | --- | --- | --- | --- | --- | --- | --- | --- |
| Upper arm | Lying down | 74.08 (14.66) | 74.39 (15.47) | -0.02 (-5.36; 5.32) | 1.61 (2.20) | 92.23 | 2.72 | 0.98 (0.97) | 1.03 (-2.16) | 0.99 (0.97-1.00) | 3.66 (3.41-3.91) |
|  | Sitting | 77.89 (14.80) | 77.97 (15.16) | -0.17 (-5.87; 5.53) | 1.89 (2.48) | 89.17 | 2.91 | 0.98 (0.96) | 0.99 (0.38) | 0.98 (0.97-1.00) | 3.73 (3.47-3.98) |
|  | Walking | 96.39 (15.98) | 96.52 (16.25) | 0.27 (-3.82; 4.35) | 1.34 (1.39) | 95.12 | 2.10 | 0.99 (0.98) | 0.99 (0.87) | 0.99 (0.99-1.00) | 2.16 (2.08-2.25) |
|  | Picking up objects | 97.01 (16.89) | 98.07 (17.30) | 1.02 (-5.02; 7.06) | 2.18 (2.34) | 87.80 | 3.25 | 0.98 (0.97) | 0.99 (1.75) | 0.98 (0.97-0.99) | 3.18 (3.01-3.35) |
|  | Jogging | 139.06 (20.51) | 139.00 (20.10) | -0.06 (-3.30; 3.17) | 0.88 (0.69) | 98.64 | 1.65 | 1.00 (0.99) | 0.98 (3.09) | 1.00 (0.99-1.00) | 1.19 (1.12-1.25) |
|  | Weight training | 129.81 (19.18) | 129.16 (17.95) | -0.65 (-13.31; 12.00) | 2.90 (2.23) | 88.15 | 6.49 | 0.94 (0.89) | 0.88 (14.72) | 0.94 (0.92-0.96) | 4.98 (4.71-5.24) |
|  | Cycling on ergometer | 145.66 (22.62) | 145.50 (22.65) | -0.16 (-2.96; 2.63) | 0.73 (0.53) | 98.99 | 1.43 | 1.00 (1.00) | 1.00 (-0.10) | 1.00 (1.00-1.00) | 0.98 (0.93-1.03) |
|  | HIIT | 174.04 (17.58) | 173.05 (17.56) | -1.00 (-7.52; 5.52) | 1.72 (1.01) | 96.26 | 3.47 | 0.98 (0.96) | 0.98 (2.28) | 0.98 (0.97-0.99) | 1.91 (1.81-2.01) |
|  | Post-exercise sitting | 102.32 (14.66) | 102.32 (14.51) | 0.00 (-2.50; 2.51) | 0.82 (0.83) | 98.89 | 1.28 | 1.00 (0.99) | 0.99 (1.48) | 1.00 (0.99-1.00) | 1.25 (1.21-1.29) |
|  | Overall | 114.67 (33.26) | 114.97 (32.96) | -0.05 (-5.84; 5.74) | 1.43 (1.35) | 94.95 | 2.95 | 1.00 (0.99) | 0.99 (1.61) | 1.00 (0.99-1.00) | 2.57 (2.53-2.61) |
| Forearm | Lying down | 74.08 (14.66) | 74.40 (15.26) | 0.00 (-5.84; 5.83) | 1.94 (2.66) | 86.26 | 2.98 | 0.98 (0.96) | 1.01 (-0.82) | 0.98 (0.97-0.99) | 4.00 (3.73-4.27) |
|  | Sitting | 77.89 (14.80) | 77.60 (15.20) | -0.54 (-7.59; 6.51) | 2.33 (3.02) | 82.26 | 3.64 | 0.97 (0.94) | 0.99 (0.60) | 0.97 (0.96-0.99) | 4.61 (4.30-4.92) |
|  | Walking | 96.39 (15.98) | 96.16 (16.50) | -0.09 (-7.25; 7.08) | 2.00 (2.11) | 90.63 | 3.66 | 0.98 (0.95) | 0.99 (0.67) | 0.98 (0.97-0.98) | 3.80 (3.65-3.95) |
|  | Picking up objects | 97.01 (16.89) | 98.93 (16.47) | 1.88 (-8.55; 12.31) | 3.63 (4.06) | 74.85 | 5.64 | 0.95 (0.90) | 0.91 (10.34) | 0.95 (0.93-0.96) | 5.49 (5.19-5.78) |
|  | Jogging | 139.06 (20.51) | 139.00 (20.06) | -0.06 (-4.42; 4.30) | 1.17 (0.92) | 96.84 | 2.23 | 0.99 (0.99) | 0.97 (3.79) | 1.00 (0.99-1.00) | 1.60 (1.52-1.69) |
|  | Weight training | 129.81 (19.18) | 127.27 (17.82) | -2.54 (-20.57; 15.48) | 5.08 (3.86) | 76.08 | 9.54 | 0.88 (0.77) | 0.82 (21.23) | 0.87 (0.84-0.89) | 7.09 (6.71-7.46) |
|  | Cycling on ergometer | 145.66 (22.62) | 145.53 (22.71) | -0.13 (-3.38; 3.17) | 0.82 (0.60) | 99.14 | 1.66 | 1.00 (0.99) | 1.00 (-0.34) | 1.00 (0.99-1.00) | 1.14 (1.08-1.20) |
|  | HIIT | 174.04 (17.58) | 170.40 (17.16) | -3.64 (-22.11; 14.83) | 4.56 (2.54) | 86.78 | 10.10 | 0.85 (0.73) | 0.83 (25.50) | 0.84 (0.81-0.86) | 5.42 (5.13-5.70) |
|  | Post-exercise sitting | 102.32 (14.66) | 100.51 (15.80) | -1.81 (-21.47; 17.85) | 2.87 (2.62) | 92.62 | 10.19 | 0.79 (0.62) | 0.85 (13.89) | 0.78 (0.76-0.80) | 9.80 (9.47-10.14) |
|  | Overall | 114.67 (33.26) | 114.11 (32.72) | -0.91 (-14.64; 12.83) | 2.72 (2.44) | 88.53 | 7.07 | 0.98 (0.96) | 0.96 (3.67) | 0.98 (0.97-0.98) | 6.09 (6.00-6.19) |

Notes. HIIT: high-intensity interval training; HR: heart rate; H10: Polar H10 heart rate monitor (reference device); SD: Standard deviation; LoA: limits of agreement; MAE: mean absolute error; MAPE: mean absolute percentage error; OLS: ordinary least squares regression; RMSE: root- mean- square error; OLS: ordinary least squares regression; CCC: concordance correlation coefficient; WSCV: within-subject coefficient of variation.
