# Supplementary material for: Wrist-Worn and Arm-Worn Wearables for Monitoring Heart Rate During Sedentary and Light-to-Vigorous Physical Activities: Device Validation Study
Source: JMIR Cardio. 2025 Mar 21;9:e67110. doi: 10.2196/67110 (PMC11951816; doi:10.2196/67110)
Supplement: Multimedia Appendix 2 [file cardio-v9-e67110-s002.docx]

**Table S1.** Accuracy and reliability results of the wrist-worn Polar Vantage V2 (dominant and nondominant wrists).

| **Vantage V2**  **wearing position** | **Activity** | **HR**  **H10 (reference)  mean (SD) [bpm]** | **HR Vantage V2 mean (SD) [bpm]** | **Systematic bias  (LoA lower; LoA upper) [bpm]** | **MAE (MAPE) [bpm]** | **5% accuracy [%]** | **RMSE**  **[bpm]** | **Pearson’s correlation coefficient or OLS  *r*-value (*r*^2^)** | **OLS**  **slope**  **(intercept [bpm])** | **CCC (95% CI)** | **WSCV (95% CI)**  **[%]** |
| --- | --- | --- | --- | --- | --- | --- | --- | --- | --- | --- | --- |
| Dominant wrist | Lying down | 74.08 (14.66) | 82.19 (26.21) | 8.11 (-30.25; 46.48) | 9.70 (12.93) | 73.36 | 21.19 | 0.67 (0.45) | 1.21 (-7.13) | 0.54 (0.48-0.59) | 26.44 (24.68-28.20) |
|  | Sitting | 77.89 (14.80) | 78.20 (14.49) | 0.31 (-16.14; 16.76) | 4.70 (6.41) | 67.59 | 8.40 | 0.84 (0.70) | 0.82 (14.50) | 0.84 (0.80-0.87) | 10.78 (10.07-11.50) |
|  | Walking | 96.39 (15.98) | 102.35 (15.08) | 5.96 (-16.09; 28.01) | 7.30 (8.80) | 63.98 | 12.73 | 0.74 (0.55) | 0.70 (35.13) | 0.69 (0.66-0.72) | 11.67 (11.23-12.12) |
|  | Picking up objects | 97.01 (16.89) | 114.24 (14.99) | 17.23 (-17.00; 51.47) | 18.42 (21.54) | 27.95 | 24.54 | 0.40 (0.16) | 0.36 (79.42) | 0.25 (0.20-0.30) | 18.01 (17.07-18.96) |
|  | Jogging | 139.06 (20.51) | 141.34 (16.33) | 2.28 (-16.41; 20.98) | 4.03 (3.84) | 85.42 | 9.81 | 0.89 (0.79) | 0.71 (42.76) | 0.86 (0.83-0.89) | 6.86 (6.50-7.22) |
|  | Weight training | 129.81 (19.18) | 131.81 (16.75) | 2.00 (-14.18; 18.18) | 5.18 (4.45) | 71.62 | 8.49 | 0.90 (0.82) | 0.79 (29.41) | 0.89 (0.87-0.91) | 6.36 (6.03-6.70) |
|  | Cycling on ergometer | 145.66 (22.62) | 144.84 (22.74) | -0.82 (-9.68; 8.04) | 1.62 (1.17) | 95.62 | 4.59 | 0.98 (0.96) | 0.99 (1.27) | 0.98 (0.97-0.99) | 3.10 (2.94-3.27) |
|  | HIIT | 174.04 (17.58) | 168.79 (16.43) | -5.26 (-25.93; 15.41) | 7.07 (4.02) | 72.63 | 11.78 | 0.81 (0.66) | 0.76 (37.06) | 0.77 (0.74-0.80) | 6.06 (5.74-6.38) |
|  | Post-exercise sitting | 102.32 (14.66) | 101.88 (15.13) | -0.44 (-7.44; 6.56) | 2.05 (2.15) | 88.70 | 3.60 | 0.97 (0.94) | 1.00 (-0.67) | 0.97 (0.96-0.98) | 3.49 (3.37-3.61) |
|  | Overall | 114.67 (33.26) | 117.60 (30.84) | 2.93 (-20.46; 26.31) | 6.07 (6.53) | 73.56 | 12.29 | 0.93 (0.87) | 0.87 (18.35) | 0.93 (0.92-0.93) | 10.41 (10.24-10.57) |
| Non-dominant wrist | Lying down | 74.08 (14.66) | 80.55 (24.38) | 6.47 (-26.97; 39.91) | 7.99 (10.46) | 75.20 | 18.25 | 0.72 (0.53) | 1.21 (-8.77) | 0.61 (0.56-0.66) | 23.04 (21.51-24.58) |
|  | Sitting | 77.89 (14.80) | 77.81 (15.36) | -0.08 (-20.09; 19.93) | 5.11 (6.88) | 66.32 | 10.21 | 0.77 (0.59) | 0.80 (15.51) | 0.77 (0.73-0.81) | 13.12 (12.25-13.99) |
|  | Walking | 96.39 (15.98) | 103.46 (13.69) | 7.07 (-17.85; 31.99) | 8.64 (10.50) | 58.93 | 14.55 | 0.64 (0.41) | 0.55 (50.42) | 0.57 (0.54-0.60) | 13.19 (12.69-13.70) |
|  | Picking up objects | 97.01 (16.89) | 114.73 (15.67) | 17.73 (-17.83; 53.28) | 18.62 (21.79) | 28.09 | 25.36 | 0.38 (0.14) | 0.35 (80.48) | 0.24 (0.19-0.29) | 18.71 (17.72-19.69) |
|  | Jogging | 139.06 (20.51) | 140.03 (17.22) | 0.96 (-15.76; 17.69) | 3.89 (3.55) | 83.69 | 8.59 | 0.91 (0.83) | 0.77 (33.53) | 0.90 (0.87-0.92) | 6.14 (5.82-6.46) |
|  | Weight training | 129.81 (19.18) | 130.61 (18.17) | 0.80 (-14.70; 16.29) | 5.22 (4.31) | 70.33 | 7.94 | 0.91 (0.83) | 0.86 (18.48) | 0.91 (0.89-0.93) | 6.09 (5.77-6.41) |
|  | Cycling on ergometer | 145.66 (22.62) | 143.74 (24.36) | -1.92 (-18.79; 14.95) | 2.81 (2.06) | 91.59 | 8.82 | 0.94 (0.88) | 1.01 (-3.04) | 0.93 (0.91-0.95) | 5.91 (5.60-6.22) |
|  | HIIT | 174.04 (17.58) | 167.66 (19.23) | -6.38 (-26.25; 13.49) | 7.89 (4.65) | 68.32 | 11.98 | 0.85 (0.73) | 0.93 (5.49) | 0.80 (0.77-0.83) | 5.83 (5.52-6.13) |
|  | Post-exercise sitting | 102.32 (14.66) | 101.70 (15.45) | -0.62 (-7.92; 6.68) | 1.96 (2.07) | 89.57 | 3.78 | 0.97 (0.94) | 1.02 (-2.99) | 0.97 (0.96-0.98) | 3.64 (3.52-3.76) |
|  | Overall | 114.67 (33.26) | 117.23 (30.78) | 2.56 (-21.88; 26.99) | 6.41 (6.82) | 71.83 | 12.73 | 0.93 (0.86) | 0.86 (18.85) | 0.92 (0.92-0.93) | 10.87 (10.7-11.05) |

Notes. HIIT: high-intensity interval training; HR: heart rate; H10: Polar H10 heart rate monitor (reference device); SD: Standard deviation; LoA: limits of agreement; MAE: mean absolute error; MAPE: mean absolute percentage error; OLS: ordinary least squares regression; RMSE: root- mean- square error; OLS: ordinary least squares regression; CCC: concordance correlation coefficient; WSCV: within-subject coefficient of variation.
